# Nontraumatic perforation of the small intestine caused by true primary enteroliths associated with radiation enteritis: a case report

**DOI:** 10.1186/s40792-021-01182-y

**Published:** 2021-04-23

**Authors:** Yusuke Hirakawa, Hirona Shigyo, Yuriko Katagiri, Kazuaki Hashimoto, Mitsuru Katsumoto, Hiroshi Tomoeda, Masahiko Nakano

**Affiliations:** Department of Surgery, Chikugo City Hospital, 917-1 Izumi, Chikugo, Fukuoka, 833-0011 Japan

**Keywords:** Enterolithiasis, True primary enterolith, Radiation enteritis, Small bowel perforation, Nontraumatic perforation

## Abstract

**Background:**

True primary enterolithiasis is an uncommon condition, and nontraumatic perforation of the small intestine (NTPSI) is also an unusual entity. Therefore, NTPSI due to true primary enteroliths is an exceptionally rare complication. Moreover, enterolithiasis and radiation enteritis are also unique combinations. Herein, we present an exceedingly rare case of NTPSI induced by multiple true primary enteroliths associated with radiation enteritis.

**Case presentation:**

A 92-year-old woman with acute abdominal pain was transferred to our hospital because a computed tomography (CT) scan performed by her family doctor revealed free air and fluid collection within her abdomen. Our initial diagnosis was upper gastrointestinal perforation, and we selected nonoperative management (NOM) with adnominal drainage. Although her general condition was stable, jejunal juice was drained continuously. Given that the CT performed 10 days after onset demonstrated perforation of the small intestine and adjacent concretion, we performed an emergency partial resection of the small intestine and jejunostomy. The resected bowel was 1 m in length and had many strictures that contained multiple enteroliths in their proximal lumens. The patient’s postoperative course was uneventful. The enteroliths were composed of deoxycholic acid (DCA). She was diagnosed with peritonitis due to NTPSI derived from multiple true primary enteroliths associated with radiation enteritis, as she had previously undergone hysterectomy and subsequent internal radiation therapy.

**Conclusions:**

Clinicians should consider the rare entity of true primary enteroliths associated with radiation enteritis in NTPSI cases with unknown etiologies.

## Background

Enterolithiasis is an uncommon medical condition and is classified into primary and secondary types [1]. Primary enteroliths are formed inside the gastrointestinal tract, whereas secondary enteroliths are introduced from outside the bowel [1]. Moreover, primary enteroliths are further subdivided into “true” and “false” subcategories [1]. True primary enteroliths are formed from normal intestinal chylus components, and false primary enteroliths are formed from ingested indigestible matter [1]. Although most primary enteroliths are false enteroliths, true primary enteroliths are extremely rare [1, 2]. On the other hand, nontraumatic perforation of the small intestine (NTPSI) is also an unusual entity [3–5]. Consequently, NTPSI caused by true primary enteroliths is an exceptionally rare complication [6]. Furthermore, enterolithiasis is rarely derived from radiation enteritis; to date, only two cases have been reported in the literature [7, 8]. The present report describes the first case of NTPSI induced by multiple true primary enteroliths associated with radiation enteritis. In addition, we discuss the clinical features, etiology, epidemiology, and pathophysiology of enterolithiasis, NTPSI, radiation enteritis, and their relevance based on the literature.

## Case presentation

A 92-year-old woman presented to her family doctor with acute abdominal pain, vomiting, and diarrhea. She was admitted to the hospital with a primary diagnosis of infectious enteritis suggested by examinations, including computed tomography (CT). The day after hospitalization, the radiologist noted free air and fluid collection within her abdomen on the CT scan from the previous day. The patient was transferred to the emergency room of our hospital for further examination and treatment.

The patient underwent brachytherapy using cobalt-60 (Co-60) gamma irradiation after hysterectomy for the treatment of uterine cancer at the age of 49. She was 145 cm tall, weighed 55 kg, and had a body mass index of 26.16.

**Fig. 1:**
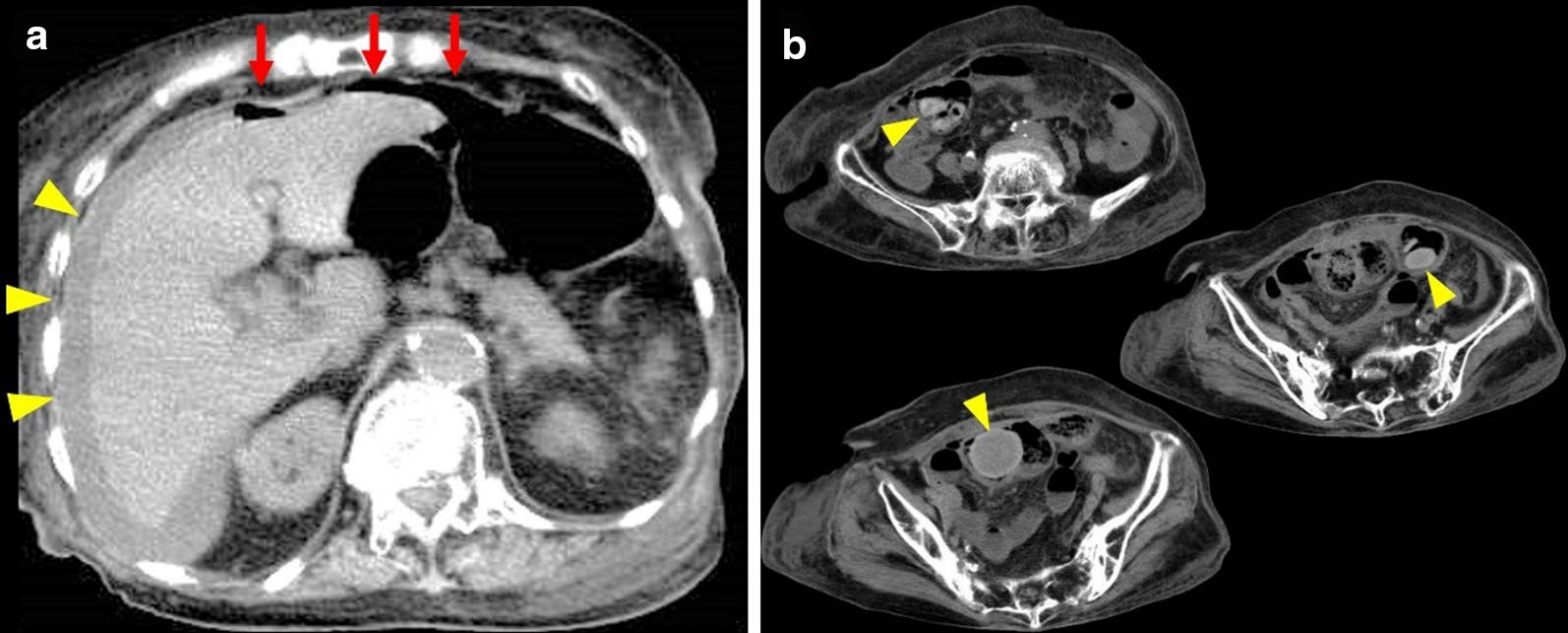
Abdominal plain computed tomography (CT) imaging performed at the first visit to a previous hospital. **a** Free air in the upper abdomen (red arrows) and fluid collection in the right subphrenic space (yellow arrowheads); **b** slightly high-density structures located in an uncertain part of the intestine (yellow arrowheads)

On admission, her vital signs were relatively stable with a blood pressure of 146/74 mmHg, heart rate of 90 beats/min, respiratory rate of 20 breaths/min, and body temperature of 36.9℃. Her abdomen was flat and had a postoperative hysterectomy scar on the lower abdominal midline. She exhibited focal tenderness, muscular guarding, and mild rebound tenderness in her lower right abdomen. The blood investigation revealed a high inflammatory response as follows: white blood cell count (WBC) of 15,500/μL, 93.5% neutrophils and C-reactive protein (CRP) level of 22.07 mg/dL. Blood urea nitrogen and creatinine levels were also increased to 37.2 and 1.3 mg/dL, respectively, whereas estimated glomerular filtration rate, hemoglobin, total protein, and albumin levels were decreased to 29.4 mL/min/1.73 m^2^, 8.2 g/dL, 4.2 g/dL, and 1.3 g/dL, respectively. Electrocardiogram and arterial blood gas results were within normal limits. Abdominal CT performed at a previous hospital showed free air mainly in the upper abdomen and fluid collection in the right subphrenic space. In addition, it confirmed the presence of multiple slightly high-density contents with spheroid or discoid shapes with sizes that varied from 1 to 4.5 cm within the intestinal tract (Fig. 1). Nevertheless, the specific organ (i.e., jejunum, ileum, or colon) in which these contents were located was not identified given the unclear boundaries of the intestinal tract in this study. The upper gastrointestinal series revealed no fistula or leakage of contrast medium in the gastroduodenal tract.

**Fig. 2:**
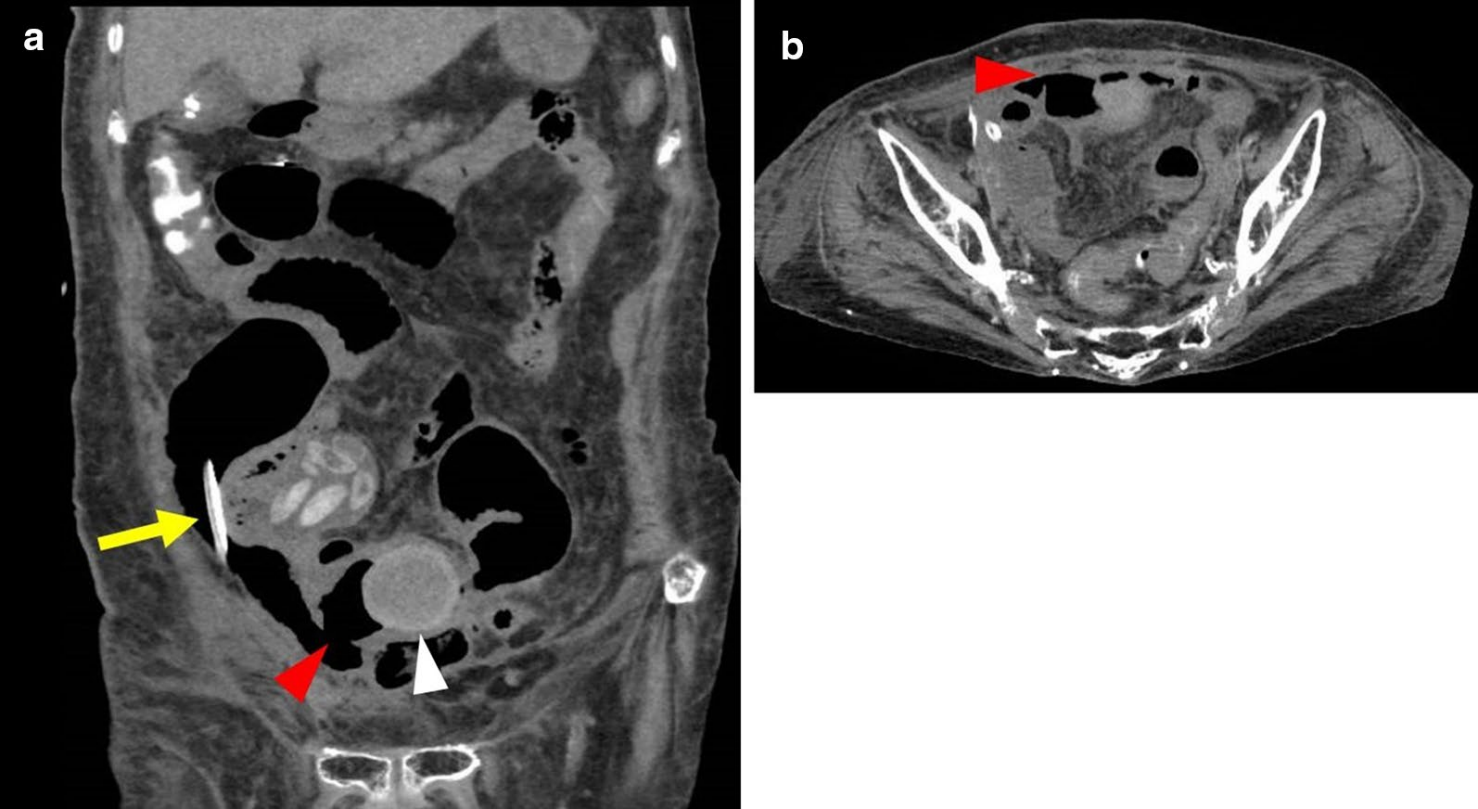
Follow-up abdominal plain CT performed 10 days after onset. **a** Coronal and **b** transaxial images reveal that the abdominal free space, including gas and the drainage tube (yellow arrow), communicates with the intraluminal space of the small intestine, which contains a slightly high-density spherical structure (white arrowhead) in the vicinity, via the perforation site (red arrowheads)

On admission, we inserted a drainage tube into the right subphrenic space, and grayish white and odorless mucus-like gastric juice was discharged from the tube. The chemical analysis of the drainage fluid revealed an alkaline phosphatase level of 332 IU/L and amylase level of 60 IU/L. Therefore, we initially suspected upper gastrointestinal perforation without continuous leakage of digestive juice. Moreover, she had stable blood pressure and no fever one day after onset. However, she was elderly and exhibited dehydration and renal failure, so the risk of surgery itself cannot be underestimated. Therefore, we started nonoperative management (NOM). After hospitalization, her vital signs remained stable, body temperature also remained normal, and abdominal pain improved. The WBC was transiently reduced to 2500 μL one day after admission, but remained within the normal range thereafter. The CRP and procalcitonin levels also exhibited peak levels of 32.5 mg/dL and 37.0 ng/mL, respectively, on the second day after admission, but both values improved thereafter. Her general condition improved day by day; however, the color of discharge from the drain turned from grayish white to dark green, similar to jejunal juice. Furthermore, the volume of drainage increased to approximately 500 mL/day. CT taken 7 days after onset showed an undrained fluid collection localized in the right paracolic gutter; nevertheless, the perforation site was not identified. Therefore, we performed additional intra-abdominal drainage and continued NOM. Thereafter, her general condition was unchanged and stable, and the volume of drainage was decreased to 200 mL/day. However, the appearance of drainage fluid remained similar to jejunal juice. An abdominal CT performed 10 days after onset demonstrated a suspicious perforated lesion as a partial defect of the small intestinal wall, and a slightly high-density structure was located immediately beside the lesion (Fig. 2). Consequently, she was preoperatively diagnosed with localized peritonitis due to NTPSI.

**Fig. 3:**
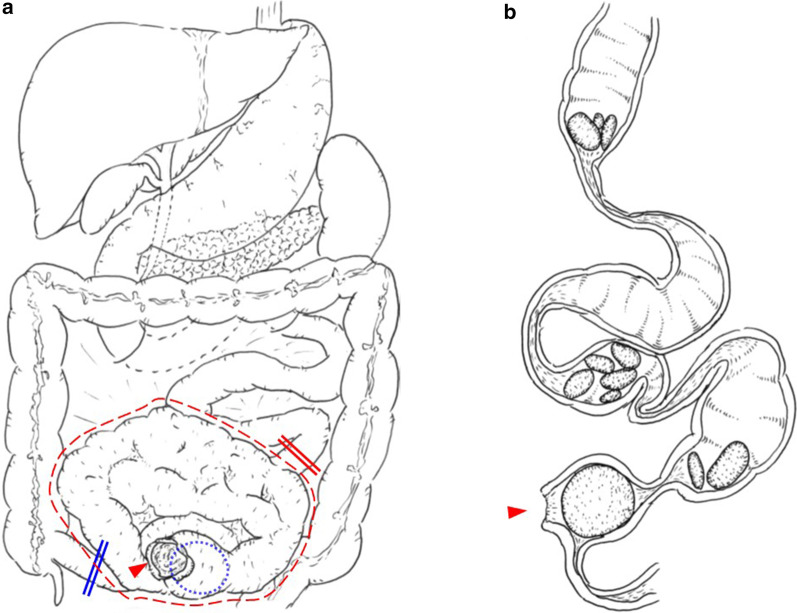
Schematic illustration of the intraoperative findings and resected specimens. **a** The severely adhered intestine, that is, the so-called “frozen pelvis” (red dashed line); intraluminal 5-cm stone (blue dotted line) near the perforation site of the small intestine (red arrowhead); proximal resection line on the anal side 1 m from the ligament of Treitz (red double line); and distal resection line on the oral side 1 m from the ileal end (blue double line); **b** The resected small intestine has many stenotic lesions and multiple stones in the prestenotic dilated lumen. The largest 5-cm stone and perforation site (red arrowhead) are located immediately on the oral side of the tightest stricture. Incidentally, the upper side is proximal, and the lower side is distal in the schematic

**Fig. 4:**
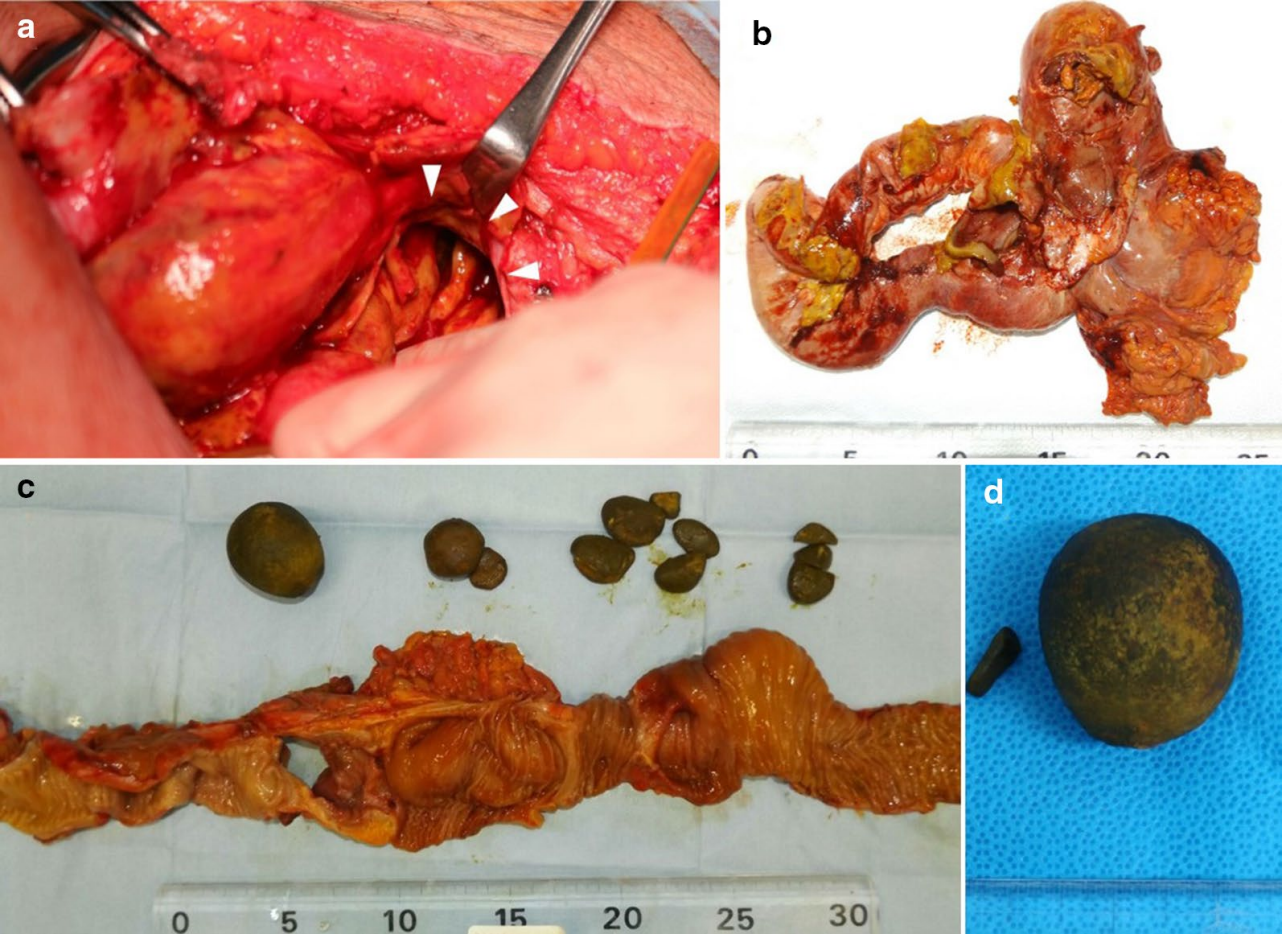
Intraoperative findings and resected specimens. a The 3-cm perforation site of the small intestine (white arrowheads); **b** the severely adhered resected small intestine; **c** the specimen after lysis and opening; **d** the largest stone is spherical and approximately 5 cm in size

We urgently performed an abdominal midline incision. The small intestine was tightly adhered to the abdominal wall and to each of the other loops of the bowel in the pelvic cavity, resulting in mass formation of the so-called “frozen pelvis”. On meticulously performing adhesiotomy and enterolysis, a 3-cm perforation appeared in a part of the small intestine. Moreover, a spherical 4.5-cm stone occupied the intestinal lumen on the immediate oral side of the perforation site. We performed en bloc resection of the mass forming sections of the small intestine from 1 m on the anal side of the ligament of Treitz to 1 m on the oral side of the terminal ileum, including the perforated lesions and stones. Additionally, end jejunostomy without ileal mucous fistulostomy was performed (Figs. 3a, 4a).

The adhered specimen of the resected small intestine was approximately 1 m in length and had a sclerosing and thickened wall of approximately the entire length. Furthermore, it exhibited many strictures and prestenotic dilatation and contained multiple deep green stones of various sizes in the proximal lumen of the strictures. The largest stone measured 4.5 cm by 4 cm and was impacted in the narrowest stenotic lumen of the resected specimen. The perforated portion was located immediately on the oral side (Fig. 3b, 4b–d).

**Fig. 5:**
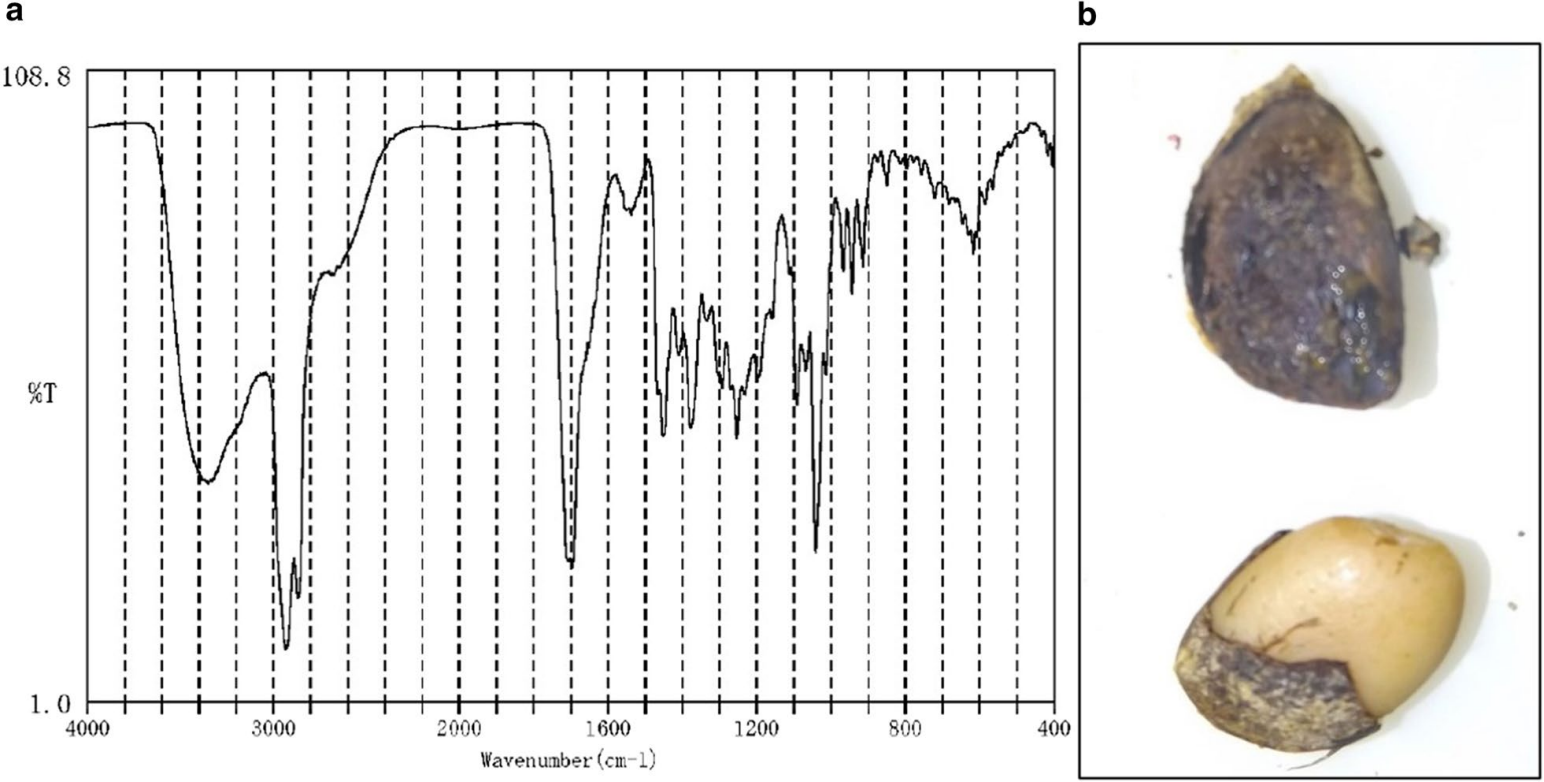
Spectrophotometric analysis and contents of the stones. **a** The absorbance pattern of deoxycholic acid (DCA) is noted; **b** some stones contain a persimmon seed-like component in their core

Infrared absorption spectrophotometric analysis revealed that the stones were true primary enteroliths composed of greater than 98% deoxycholic acid (DCA) (Fig. 5a). Some discoid stones were laminated and contained a structure suspected to be a persimmon seed in the core (Fig. 5b). The histopathological findings of resected specimens of the small intestine revealed severe fibrosis and hyalinization in the layers from the muscularis propria to the serosa compatible with the diagnosis of radiation enteritis. The final diagnosis of the present case was peritonitis due to NTPSI derived from multiple true primary enteroliths associated with radiation enteritis.

In this patient, surgical site infection of the wound occurred 4 days after operation; therefore, she underwent wound lavage and negative pressure wound therapy. She resumed oral ingestion of food 19 days after the operation. Her postoperative progress was almost uneventful; however, several days were required for wound healing and improvements in daily living activities because she was frail and elderly. She was transferred to a nearby hospital 104 days after the operation.

## Discussion

Enterolithiasis is the presence of an enterolith within the intestinal lumen and is a rare condition; its prevalence in selected populations varies widely from 0.3% to 10% because it is largely dependent upon clinical presentation, etiology, and underlying risk factors [1, 6]. Enteroliths are defined as endogenous foreign bodies in the gastrointestinal tract [2]. Enteroliths are classified into primary and secondary types [1]. Primary enteroliths are defined as concretions that develop inside the bowel. In contrast, secondary enteroliths form outside the gastrointestinal tract and migrate into the bowel accompanied by fistulation to the intestinal lumen, which is similar to that observed in cholelithiasis or urolithiasis [1]. Furthermore, primary enteroliths are subdivided into “true” and “false” subcategories [1, 2]. Clinically, true enteroliths are extremely rare, whereas false enteroliths are much more common in primary enteroliths [1, 9–11]. False primary enteroliths are composed of orally ingested indigestible matter, such as feces (fecaliths), hair balls (trichobezoars), vegetable matter (phytobezoars) or other exogenous substances, such as varnish and barium sulfate [1, 6]. Conversely, true primary enteroliths are defined as stones made of precipitants and/or deposits that normally exist in the intestine, such as choleic acids and calcium salts [1, 2]. The composition of true primary enteroliths is closely related to their location. Choleic acids precipitate under lower pH conditions, so enteroliths formed in the duodenum and jejunum are mostly composed of choleic acid, which is largely affected by significant diverticular disease, strictures, or stenosis [2, 6]. In contrast, calcium salts are soluble in water and acidic environments; thus, they precipitate under alkaline pH conditions in the distal small intestine or colon, most commonly in the terminal ileum [2]. With regard to the mechanisms contributing to the formation of enteroliths, two factors are considered. The first comprises mechanical factors, such as intestinal stasis due to intestinal diverticular diseases [12–15]; blind pouches after side-to-side enteroanastomosis [16, 17]; blind loops including the afferent loop in Billroth-II gastrojejunostomy and Roux-en-Y reconstruction [18–21]; incarcerated hernias [8]; intestinal kinking from intra-abdominal adhesions [8]; and intestinal strictures and stenosis encountered in infectious and inflammatory conditions, such as Crohn’s disease [8], ulcerative colitis [8], intestinal tuberculosis [22, 23], and, rarely, radiation enteritis [7, 8]. By contrast, the second factor involves chemical properties, such as acidic conditions. Impaired intestinal flow and stasis in the gastrointestinal tract may promote microbiome overgrowth-accelerated changes in the pH of the surrounding bowel contents, resulting in precipitation of substances out of solution [6]. In our case, the concretions were almost completely composed of DCA, that is, true primary enteroliths of the choleic acid type, and the location was the distal jejunum (as in many reports). The radiological diagnosis of enteroliths depends on their calcium content [8]. Enteroliths containing a higher proportion of calcium salts are more radiopaque and form in the relatively more alkaline environment of the distal ileum. Choleic acid enteroliths are more radiolucent and form in the more acidic environment of the proximal small intestine [6]. In the present case, the enteroliths were radiolucent, were not detected on abdominal X-ray, and appear as slightly high-density matter exclusively on abdominal CT. Only a few studies have reported that true primary enteroliths may occasionally have a central “fruit pit”, such as that from a plum [1, 2, 6]. Some enteroliths of the present case also contained a fruit seed-like component in their nucleus. These pits were presumed to be persimmon seeds based on their appearance, which is exceptionally unique. Upon our inquiry, the patient reported no memory of ingesting persimmon seeds in the past.

Radiation enteritis is a rare cause of enterolithiasis. A literature search of the PubMed database analysis yielded 4 results published between 1962 and 2020 when searching for “enterolith AND radiation enteritis”. Excluding nonrelevant results and limiting the scope to articles written entirely in English, only 2 reports describing enterolithiasis associated with radiation enteritis were identified [7, 8]. In our case, there were multiple stenotic lesions derived from histopathologically evidenced radiation enteritis in the jejunum, and several true primary enteroliths had been fixed in each lumen immediately proximal to the strictures. These findings suggest that the stagnation of intestinal flow leading to the formation of enteroliths was caused by the thickening and narrowing of the small intestine associated with radiation enteritis. Even in this respect, the present case is extremely rare. In addition, the finding of “frozen pelvis” is often caused by subacute or chronic generalized peritonitis, including the result of NOM; however, in our case, we hypothesized that the frozen pelvis was derived from radiation therapy in the past because the fluid collection did not spread throughout the entire abdominal cavity but localized to the right paracolic gutter from the beginning of onset.

**Fig. 6:**
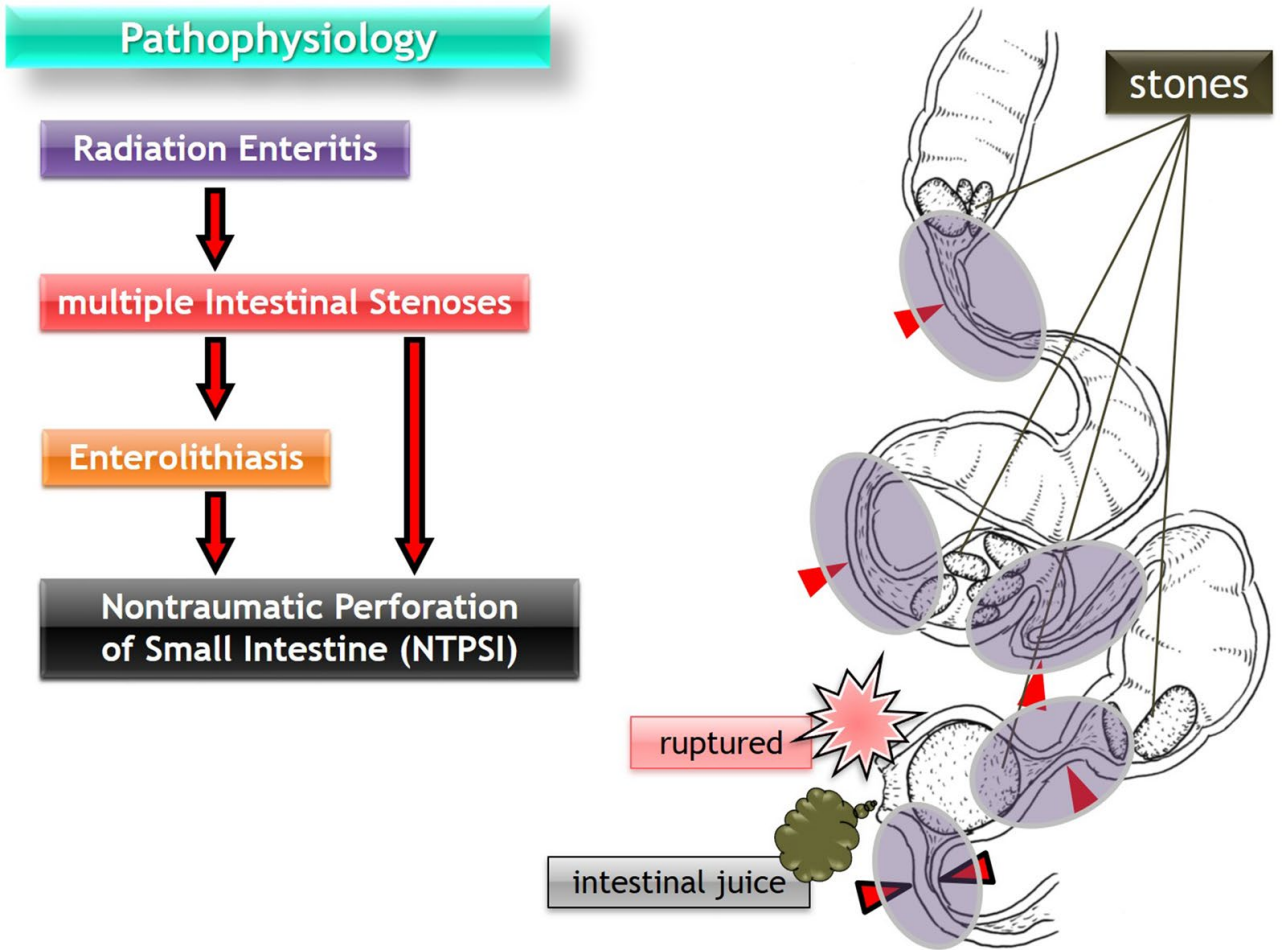
Pathophysiological chart of the present case. Initially, radiation enteritis (purple shadowed circles) causes multiple strictures (red arrowheads) in the small intestine. Second, multiple true primary enteroliths arise in the prestenotic area as a result of intestinal stasis. Finally, the narrowest stenotic lesion (red arrowheads bordered in black) itself or impaction of the enterolith in the stricture causes nontraumatic perforation of the small intestine (NTPSI)

Small bowel obstruction is a common surgical presentation. Nevertheless, as Nonose et al. [24] described only 41 cases reported up to 2011, primary jejunal enterolithiasis uncommonly results in mechanical obstruction of the small intestine, which is known as enterolith ileus or pseudogallstone ileus. In the present case, it is reasonable to hypothesize that true primary enteroliths caused ileus prior to small intestinal perforation, which is also rare. Consistent with this concept, NTPSI, which is defined as small intestinal perforation excluding perforated duodenal ulcers and perforation due to external hernias and primary ischemic events, is a rare entity with an incidence reported in the literature of one case per year per 300,000 to 350,000 individuals [3–5]. Accordingly, NTPSI due to enteroliths has been occasionally reported, but is an exceedingly rare complication; thus, the incidence is difficult to quantify [6]. The reported causes of enteroliths resulting in NTPSI are mainly duodenal diverticulum [25], Meckel’s diverticulum [12], jejunal diverticulum [26, 27], and Crohn’s disease [28], but there are no reports relevant to radiation enteritis. Thus, to the best of our knowledge, this is the first case of NTPSI caused by multiple true primary enteroliths associated with radiation enteritis, and the hypothesized pathophysiological chart is shown in Fig. 6.

The mortality of NTPSI was reported to range from 15 to 39% in the early era because surgery was often delayed due to difficulties experienced with the initial diagnosis of the precise cause and site of perforation [3–5, 29]. However, the recent literature has demonstrated that the mortality rates might be lower than those in the past [3]. One reason may be the development of modern imaging modalities, particularly CT. CT imaging currently substantially aids the evaluation of a suspected perforation and is demonstrated to be accurate in defining the possible site of the perforation [5]. Once NTPSI is diagnosed, urgent surgical intervention is typically required [3–5, 30]. Jain et al*.* [30] mentioned that the appropriate operative procedure for NTPSI depends upon the presence or absence of adverse conditions in the patient, such as hypotension, a long perforation-to-operation interval, a high volume of peritoneal contaminant fluid, and/or an edematous bowel not available for repair. For patients with adverse factors, the no suture line-in procedure, such as ileostomy, seems to be better [30]. In our case, localization of fluid collection induced by frozen pelvis and intra-abdominal drainage repressed the typical symptoms of peritonitis and stabilized her general condition. Therefore, we continued NOM given that we judged a low need for surgery, and we could not deny that the NOM alone may cure her condition. Furthermore, we hesitated to perform surgery because we could not identify the accurate perforation site and we could not deny the possibility of duodenal perforation and leakage of refluxed jejunal juice via the perforation site. Consequently, the time between onset and surgery was 11 days. This finding is consistent with the difficulty of preoperative diagnosis of NTPSI, as reported in previous studies [3–5, 29]. As a result of performing CT up to five times, an accurate diagnosis of NTPSI was finally made. A large perforation site and an incarcerated stone hypothesized to be the cause of perforation were identified; therefore, we decided to perform an ileostomy for this patient given the presence of adverse factors, such as a long interval between onset and operation, a large amount of intraperitoneal contamination, and small intestinal edema.

## Conclusions

Clinicians should consider small bowel perforation as a differential diagnosis when perforated lesions cannot be identified accurately in patients with intra-abdominal free air. In addition, if a patient with suspected NTPSI has slightly to very high-density components in the small bowel on CT, it is crucial to consider that enterolithiasis, a rare entity, can be a cause of intestinal perforation. Furthermore, if such a patient has a past history of undergoing radiation therapy (such as brachytherapy) to the abdomen, radiation enteritis-associated enterolithiasis must also be considered.

## Data Availability

Not applicable.

## Abbreviations

NTPSI: Nontraumatic perforation of the small intestine
CT: Computed tomography
NOM: Nonoperative management
DCA: Deoxycholic acid
Co-60: Cobalt-60
CRP: C-reactive protein
UDCA: Ursodeoxycholic acid
